# Mechanism- and Data-Informed Hierarchical Bayesian Electro-Optical Reconnaissance Performance Evaluation

**DOI:** 10.3390/s26216894

**Published:** 2026-10-30

**Authors:** Weicong Ma, Fulun Peng, Jing Wang, Xiaotian Wang, Peng Xie, Yongcong Zhang

**Affiliations:** Xi’an Institute of Applied Optics, Xi’an 710065, China; matrixgardener123@gmail.com (W.M.);

**Keywords:** electro-optical reconnaissance, performance evaluation, hierarchical Bayesian modeling, mechanism–data dual drive, uncertainty quantification

## Abstract

Performance evaluation of electro-optical reconnaissance systems is affected by multi-source uncertainty, data heterogeneity, and model mismatch. This paper presents a hierarchical Bayesian evaluation framework that combines physical constraints with data from different sources. The capability model represents detection, recognition, tracking, and localization through four bounded indicators. Physical relations inform the prior structure, while observation, parameter, and hyperparameter layers represent variation within and between data sources. Joint modeling describes the dependence among the indicators and provides posterior predictions with uncertainty intervals. The reported comparisons examine prediction error, robustness across scenarios, uncertainty calibration, and performance at different sample sizes. These results illustrate the use of a common probabilistic framework for comparing capability estimates and their uncertainty.

## 1. Introduction

Electro-optical reconnaissance has become an important tool for acquiring strategic and tactical intelligence in regional conflicts. The principle of this technology is to identify and track targets by detecting the electromagnetic waves they reflect or radiate. It switches transmission and reception frequency bands and calculates the physical coordinates of the target. The main advantages of the technology are that it is non-contact, highly resistant to electronic jamming, capable of operation day and night, and adaptable to a variety of platforms. These features support integrated sensing across different land, sea, and air systems.

In operational environments, such systems are constantly subjected to various environmental interferences such as atmospheric turbulence, changes in visibility, background clutter, and electromagnetic interference. Uncertainties at the system level due to equipment parameters such as platform vibration, axis drift, time-synchronization errors, calibration inaccuracies, and computational constraints make these situations even more challenging. These factors lead to large spatiotemporal variations in the performance of core functions like detection, recognition, tracking, and positioning. Traditional static performance evaluations carried out in ideal or semi-ideal conditions usually depend on a very limited number of performance indicators and low-dimensional data sources, which complicates reliable extrapolation and generalization of evaluation results. This limitation restricts the use of these evaluations for mission planning, equipment resource scheduling, payload selection, and field acceptance, where the results should be measurable and interpretable, with uncertainty bounds.

Research at the mission level of electro-optical/infrared reconnaissance platforms spans the technical chain from target localization and error correction to coverage and stabilization control, as well as computational imaging and reconstruction. Bai et al. [1] combined digital elevation models with multi-source sensors to improve geographic coordinate inversion under complex terrain conditions. Their framework uses detected pixel coordinates to construct the target line-of-sight vector. This vector is then jointly solved with the aircraft and payload measurements. The positioning errors for the primary and secondary targets were 16.47 m and 27.36 m, respectively, when the experiments were carried out at an approximate flight altitude of 3000 m. After enhanced filtering, these errors were approximately 7 m and 11 m. Chen et al. [2] proposed installing an inertial navigation system (INS) on the electro-optical payload and using main INS data for motion-based alignment to eliminate systematic errors caused by payload–platform coupling. Vehicle and airborne experiments demonstrated that the azimuth error converged very quickly, to around 0.1° at 1σ, and that the ground localization accuracy was improved to about 6 m. Chen et al. [3] modeled pointing errors between the imaging plane and the equipment-base coordinate system and conducted Monte Carlo sensitivity analysis to quantify the main error sources and prioritize calibration.

For geoscanning coverage and stabilization control, Ren et al. [4] tackled the problem of image-stitching failures and disturbance suppression through an integrated method. They standardized the coordinate systems and transformation relationships, and introduced compensation angular-velocity commands into the roll and pitch scanning of the gimbal. This approach enabled seamless coverage of ground areas, demonstrating its effectiveness for continuous geospatial scanning. Li et al. [5] developed a 3D sector-scanning coverage mechanism and a corresponding implementation scheme for unattended continuous surveillance, which includes technologies for optimized moving-target detection, false-alarm control, and field-of-view adaptation. Their experiments showed simultaneous improvements in target detection probability and early-warning accuracy. Fang et al. [6] combined camera geometric modeling and image motion compensation using INS-derived attitude angles together with intrinsic calibration and multi-coordinate-system consistency processing to reduce image displacement caused by attitude changes and velocity instability. The method improved image stability and reconstruction quality for downstream stitching and mapping.

Research into computational imaging has enhanced performance capabilities of electro-optical reconnaissance systems at the device-reconstruction level. Cao et al. [7] suggested a pseudo-double-layer odd-even microlens combination on the segmented planar imaging detector for electro-optical reconnaissance, SPIDER, to reduce the loss of spatial-frequency information. End-to-end numerical simulations indicated improvements in peak signal-to-noise ratio and detail preservation. Lv et al. [8] showed through integrated optical-interference simulations that, with the increase in the number of interference arms and the baseline length, combined with baseline-matching optimization, low-frequency sampling rates and overall imaging quality can be improved. They identified the optimum imaging distance and phase-noise tolerance and gave parametric design guidance for engineering implementation of SPIDER-like systems. These studies address complementary aspects of electro-optical imaging: localization and stabilization concern acquisition and geometric consistency, whereas computational reconstruction concerns the information retained by the imaging system.

Research on performance evaluation has also examined Bayesian modeling. Dai Qin and his colleagues [9] applied Bayesian networks to remote-sensing data classification and showed how effective probabilistic graphical models can be for fusion of multi-source heterogeneous data. Liu Wenbo and his colleagues [10] created a Bayesian-network-based model to determine the threat level of reconnaissance which allows probabilistic inference and quantitative classification of threat elements. Song Xudong and his colleagues [11] performed the target-tracking method’s performance characterization in a Bayesian framework and gave the theoretical basis for the probabilistic assessment of tracking capability. Sun Yanfei and his colleagues [12] evaluated the electro-optical reconnaissance equipment’s countermeasures effectiveness while dealing with performance metrics under complex countermeasures. The use of Bayesian networks has extended to system-level performance evaluation. Yu Xiaolan and his colleagues [13] applied Bayesian networks to the operational effectiveness analysis of aerospace reconnaissance equipment and have established the multi-level inference framework that links missions, capabilities and indicators, which is now widely used in the field. Zhang Mengqi and his colleagues [14] introduced a Bayesian-network-based method for system performance evaluation, which allows probabilistic quantification of complex system performance in the context of data-intelligence integration. These studies provide a basis for Bayesian methods in the performance evaluation of electro-optical reconnaissance systems.

Physics-informed Bayesian modeling and multi-source data fusion provide the methodological context for this study. Physical relations can inform prior assumptions, while hierarchical structure represents differences in noise and coverage among simulation, laboratory, field, and historical data. The present framework combines bounded capability functions, physics-informed prior structure, and joint modeling of four indicators. Its uncertainty analysis distinguishes observation noise, model-parameter uncertainty, scenario differences, and data-source differences.

The framework combines information from imaging, MTF, and SNR relations with a likelihood for observations from heterogeneous data sources. Observation, parameter, and hyperparameter layers describe the corresponding levels of variation. The joint model uses detection, recognition, tracking, and localization as its four capability indicators, with shared variables and covariance structure representing their dependence. Posterior predictions and uncertainty intervals provide a common basis for reporting these indicators.

## 2. Electro-Optical Reconnaissance Capability Model and Data Acquisition System

### 2.1. Construction of Electro-Optical Reconnaissance Capability Model

#### 2.1.1. Capability Model

The electro-optical reconnaissance capability model comprises four interrelated components:

(1) Detection Capability: Detecting targets under complex conditions depends mainly on signal-to-noise ratio (SNR) and atmospheric conditions [15], as well as background clutter:

(1) $$P_{d}=f_{\det}(SNR,\sigma_{clutter},\tau_{atm},\theta_{t\arg et})$$

$P_{d}$ denotes the probability of detecting a signal when it is present. SNR describes signal strength relative to the noise background. $\sigma_{\mathrm{clutter}}$ is the standard deviation of the background clutter, representing interference intensity. $\tau_{\mathrm{atm}}$ is the atmospheric transmittance, ranging from 0 to 1, which quantifies the attenuation of optical signals due to atmospheric interference. $\theta_{\mathrm{target}}$ is the target’s apparent angular size, expressed in milliradians (mrad), and reflects the angular resolution of the target on the imaging plane.

For the numerical implementation, *f*_det_ is a bounded logistic mapping on the linear-scale SNR: *z*_d_ = 0.35 ln(SNR_lin_ + *ε*) + 1.2 × *τ*_atm_ + 0.15 × *θ*_target_ − 2.0 × *σ*_clutter_ − 0.8; *P*_d_ = clip(logistic(*z*_d_), 0.10, 0.99), where SNR_lin_ = $10^{{SNR}_{dB}/10}$. The coefficients and clipping bounds specify the function used in the implementation.

(2) Recognition Capability: Recognition of a detected target depends on image quality and target characteristics [16], as well as algorithm performance.

(2) $$P_{r}=f_{rec}(MTF,\sigma_{noise},\phi_{t\arg et},\alpha_{a\mathrm{lg}o})$$

In Equation (2), $P_{r}$ stands for the probability of unambiguously recognizing a target; MTF is the modulation transfer function at the spatial frequency corresponding to the target features (ranging from 0 to 1), and describes the preservation of target detail by the imaging system; $\sigma_{\mathrm{noise}}$ is the noise standard deviation corresponding to image noise, consisting of readout noise and quantum noise, among others; $\phi_{t\arg et}$ is the inter-class separation difficulty coefficient; larger values indicate smaller differences between classes and greater recognition difficulty; and $\alpha_{\mathrm{algo}}$ is the coefficient related to the recognition algorithm’s performance, which broadly characterizes its accuracy and tolerance to interference in classification.

The recognition function is *z*_r_ = 2.5 × MTF + 1.0 × *α*_algo_ − 1.8 × *σ*_noise_ − 1.2 × *ϕ*_target_ + 0.2; *P*_r_ = clip(logistic(*z*_r_), 0.05, 0.95). Here, *ϕ*_target_ denotes the inter-class confusion coefficient and *α*_algo_ denotes the recognition-algorithm coefficient.

(3) Tracking Capability: This is the ability to maintain continuous tracking of moving targets [16]. It is influenced by target motion characteristics, platform stability [4], and algorithmic robustness [17]:

(3) $$P_{t}=f_{track}(v_{t\arg et},\sigma_{platform},\beta_{a\mathrm{lg}o},\Delta t)$$

The tracking retention probability is denoted by $P_{t}$; the target velocity $\left(m\cdot s^{-1}\right)$ is denoted by $v_{t\arg et}$ and reflects the maneuver intensity of the target; $\sigma_{platform}$ is the standard deviation of the platform pointing stability error (mrad); $\beta_{a\mathrm{lg}o}$ is the performance coefficient of the tracking algorithm, which characterizes the effect of platform jitter on tracking accuracy; and Δt is the frame interval (s), which affects the accuracy of motion prediction.

The tracking function is *z*_t_ = 0.8 × *β*_algo_ − 0.12 × *v*_target_ − 1.5 × *σ*_platform_ − 0.5 × Δ*t* + 1.0; *P*_t_ = clip(logistic(*z*_t_), 0.20, 0.98), with *v*_target_ in m/s, *σ*_platform_ in mrad, and Δ*t* in s.

(4) Localization Capability: Determining the spatial position of a target depends on geometric accuracy and calibration [18], as well as measurement accuracy [19]:

(4) $$P_{l}=f_{loc}(\sigma_{geo},\sigma_{cal},\sigma_{meas},d_{t\arg et})$$

In Equation (4), $P_{l}$ denotes the localization capability; $\sigma_{geo}$ represents the quality of the geometric configuration; $\sigma_{cal}$ is the system calibration error; $\sigma_{meas}$ denotes measurement noise, and $d_{t\arg et}$ is the target distance in meters.

The localization function maps the error-budget terms to a bounded score: *e* = *σ*_geo_ + *σ*_cal_ + *σ*_meas_; *z*_l_ = 1.5 − 3.0 × *e* − 0.0008 × *d*_target_; *P*_l_ = clip(logistic(*z*_l_), 0.30, 0.99). The distance variable *d*_target_ is expressed in meters. *P*_l_ denotes the localization capability score used in the joint evaluation, rather than a distance error measured in meters.

Input descriptors. The four capability functions use the fields listed in the input table. Detection depends on SNR, atmospheric transmittance, target angular size, and clutter intensity. The recognition inputs are MTF, image noise, inter-class confusion, and the recognition-algorithm coefficient. Target velocity, platform pointing stability, the tracking-algorithm coefficient, and the frame interval describe tracking conditions. Localization uses the geometric, calibration, and measurement terms together with target distance. Symbols and units are defined with Equations (1)–(4).

#### 2.1.2. Capability Model Validation

The simulation environment comprises 1000 representative scenarios generated by Latin Hypercube Sampling (LHS). Each scenario is generated through uniform sampling over the key parameter space. The sampled parameters and ranges are: signal-to-noise ratio SNR∈[3,30]dB, atmospheric transmittance $\tau_{atm}\in [0.3,0.95]$, target apparent angle $\theta_{t\arg et}\in [0.5,10]$ mrad, clutter standard deviation $\sigma_{clutter}\in [0.01,0.5]$, and platform stability error $\sigma_{platform}\in [0.05,1.0]$ mrad. For each parameter set, the capability indicators predicted by Equations (1)–(4) are compared with the Monte Carlo benchmark values. This comparison characterizes the numerical agreement with the simulation benchmark and the response of the indicators to changes in the input parameters. Table 1 summarizes prediction and interval-calibration metrics across the 1000 scenarios. Table 2 lists the sensitivity rankings from Sobol variance decomposition.

In the simulation-based comparison (Table 1), the prediction interval coverage probability is 0.948 for a nominal 95% interval, with a mean interval width of 0.186. The expected calibration error is 0.021.

The sensitivity analysis in Table 2 ranks SNR as the strongest factor, followed by atmospheric transmittance $\tau_{atm}$, the ratio between the target feature and the contrast $\theta_{t\arg et}$, background clutter intensity $\sigma_{clutter}$, and the platform pointing stability $\sigma_{platform}$. The ranking indicates the influence of signal quality and transmission conditions on overall system performance. Background clutter intensity and atmospheric transmittance represent different environmental factors. Atmospheric transmittance is related to the energy of the signal that is lost mainly through absorption and scattering as it travels along the path, whereas background clutter intensity depends on the complexity of the terrain and the infrared radiative properties of the area where the target is located. Their different mechanisms support treating these factors separately and modeling them independently.

The semi-physical evaluation combines real electro-optical components with simulated targets, atmospheric attenuation, and controlled backgrounds. The reported design contains 200 combinations of five signal-to-noise ratio levels, four atmospheric-transmittance conditions, five target types, and two background-complexity levels. Table 3 summarizes the comparison between the model and the laboratory measurements, including prediction error, bias, and prediction-interval coverage.

The 500 laboratory samples comprise 200 controlled-condition runs and 300 repeat measurements under a subset of 60 conditions. All 500 samples are included in the 4500-sample corpus. The separate 1000-scenario LHS validation set contains simulated scenarios and does not include these laboratory repeats.

The semi-physical results in Table 3 give a correlation coefficient of 0.92, an RMSE of 0.129, and an MAE of 0.101. The mean bias is −0.006. For the nominal 95% prediction intervals, PICP is 0.943 and PIW is 0.194.

### 2.2. Multi-Source Data Acquisition and Quality Assurance

#### 2.2.1. Multi-Source Data Acquisition Scheme

The corpus contains 4500 samples from four source categories: 3000 physics-simulation samples, 500 semi-physical laboratory samples, 200 field samples, and 800 historical samples. The source labels distinguish observations obtained under different acquisition conditions. This grouping is retained in the hierarchical analysis so that the sources are not treated as interchangeable.

Table 4 summarizes the allocation of the 4500 samples by source and partition. The 1000 LHS scenarios used for simulation-based validation in Section 2.1.2 are separate from this corpus; their role is to compare the capability functions with the simulation benchmark.

#### 2.2.2. Data Quality Assurance Mechanism

Data quality is considered in terms of field completeness, consistent variable definitions, valid numerical ranges, and source label consistency. A common schema supports comparison among the four source categories.

The input and source label tables are paired by row order. The labels identify simulation, laboratory, field, and historical samples and are distinct from the four capability outputs.

Field data refers to the 200 samples assigned to the outdoor-acquisition category, whereas historical records form a separate category of 800 samples. The test partition contains 467 simulation, 33 laboratory, 25 field, and 175 historical samples, forming a mixed-source test set.

## 3. Hierarchical Bayesian Evaluation Model Driven by Mechanism and Data

### 3.1. Construction of Mechanism-Informed Priors Based on the Capability Model

#### 3.1.1. Capability-Constrained Priors

The capability model in Section 2 is accompanied by boundary, inter-capability, and task-related constraints. These assumptions define the structure used for inference.

The capability boundary constraint specifies the numerical ranges used for the four bounded indicators: [0.10, 0.99] for detection, [0.05, 0.95] for recognition, [0.20, 0.98] for tracking, and [0.30, 0.99] for localization. These bounds are part of the model specification:

(5) $$P_{d}\in [0.1,0.99],P_{r}\in [0.05,0.95],P_{t}\in [0.2,0.98],P_{l}\in [0.3,0.99]$$

The inter-capability component describes joint variation among the four indicators. Detection, recognition, tracking, and localization depend on different input factors, but they may also vary together because of shared imaging conditions and platform state. Equation (6) states the joint-distribution specification and the ordering restrictions adopted in this model. These restrictions are modeling assumptions rather than a universal physical hierarchy among the four capabilities.

(6) $$z_{i}∼N(\mu_{p},\Sigma_{p}/\lambda ),\;subject\;to\;P_{r}\leq P_{d},P_{t}\leq P_{r},P_{l}\leq P_{t}$$

The mean vector and covariance matrix characterize the central values and joint variation in the capability indicators. The ordering restrictions remain part of the specification in Equation (6).

The task-requirement constraint represents a specified minimum capability threshold. Different reconnaissance missions impose different requirements on detection, recognition, tracking, and localization. Mission accomplishment depends on meeting the corresponding performance thresholds. The joint performance constraint requires the minimum of the capability indicators to exceed the task-specific threshold $P_{threshold}$. The threshold is an assumption of the evaluation setting.

(7) $$\min(P_{d},P_{r},P_{t},P_{l})\geq P_{threshold}$$

These constraints define the boundaries, dependence assumptions, and task threshold used in the capability model. They make the assumptions of the evaluation explicit.

#### 3.1.2. Physics-Informed Priors

Physics-informed priors are used to align the capability model with the physical principles of imaging, transmission, and noise. The priors address three aspects:

(1) Imaging-chain prior: The modulation transfer function (MTF) describes the transfer characteristics of an imaging system [20] and its ability to retain target information at different spatial frequencies [21]. We use a Gaussian approximation for the transfer function of a diffraction-limited optical system, combined with an exponential attenuation term for motion-blur degradation. In a cascaded system, the overall MTF is the product of the MTFs of the individual stages [22]. Accounting for the optical diffraction limit and motion blur gives the following expression:

(8) $$MTF(f)=\exp(-\frac{f^{2}}{2\sigma_{optics}^{2}})\cdot \exp(-\frac{f^{2}}{2\sigma_{motion}^{2}})$$

Here, f represents spatial frequency, $\sigma_{optics}$ denotes the optical system’s resolution capability, which depends on factors such as aperture and aberrations, and $\sigma_{motion}$ represents the attenuation of image sharpness due to motion blur. This model maintains the physical interpretability of image quality.

(2) Signal-to-noise ratio prior: The signal-to-noise ratio (SNR) [23] measures the detectability of a target signal in the presence of noise. The SNR decomposition model uses the signal variance fraction (SVF). This quantity describes the proportion of total observed variance contributed by the effective target signal:

(9) $$SVF=\frac{S_{signal}^{2}}{S_{signal}^{2}+S_{noise}^{2}+S_{dark}^{2}}$$

We use $S_{signal}$ to denote target signal intensity, while $S_{noise}$ denotes system noise from sensor readout, and $S_{dark}$ denotes dark-current noise. This relation links detection performance to the optical system and the detector’s physical properties, supporting the model’s robustness and physical plausibility.

(3) Atmospheric transmission prior: Atmospheric transmission affects electro-optical reconnaissance performance. An approximate solution to the radiative transfer equation [24] is used to model atmospheric transmittance $\tau_{atm}$:

(10) $$\tau_{atm}=\exp\left(-\int_{0}^{R}a(r)dr\right)$$

The term a(r) denotes the sum of the atmospheric absorption and scattering coefficients along the propagation path at a distance r. R is the propagation distance between the target and the sensor. The model represents the attenuation of electromagnetic waves under meteorological conditions involving humidity, aerosol concentration, and visibility, allowing it to describe varied environments.

### 3.2. Hierarchical Bayesian Model Structure

#### 3.2.1. Observation-Layer Modeling

The observation layer represents observations from heterogeneous sources within a common statistical structure. It uses the four bounded capability indicators defined in Section 2: detection, recognition, tracking, and localization. Source-dependent noise accounts for differences among the source groups. The observation model is written as follows:

(11) $$y_{i}|\theta ,s_{i}∼N(\mu (\theta ),\sigma_{s_{i}}^{2})$$

The observation i is denoted by $y_{i}$, and the capability model parameters by θ. The four data sources—simulation, laboratory, field, and historical data—are represented as $s_{i}\in \{1,2,3,4\}$. The mean function μ(θ) represents the typical response of the capability model to the observed data. The term $\sigma_{s_{i}}^{2}$ specifies the noise level associated with each data source. Simulation data usually have low noise but can be affected by model mismatch; field data are noisier and more closely reflect real conditions. This formulation enables data from different sources to be compared within a unified probabilistic framework.

#### 3.2.2. Parameter-Layer Modeling

The parameter layer characterizes variation in capability and system parameters across scenarios. The formulation decomposes each parameter into a shared baseline and a scenario-specific offset:

(12) $$\theta_{j}=\theta_{base}+\Delta_{j}$$

Here, $\theta_{j}$ denotes the parameter value for scenario j. $\theta_{base}$ represents the globally shared baseline parameter, and $\Delta_{j}$ denotes the scenario-specific offset. This formulation preserves parameter consistency across scenarios while capturing the influence of environmental variation on capability parameters. Under field conditions with reduced visibility, $\Delta_{j}$ is adjusted to account for degradation within the imaging chain.

#### 3.2.3. Hyperparameter-Layer Modeling

The hyperparameter layer represents variation among the modeled groups and supports information sharing within the hierarchy. Its statistical form is:

(13) $$\Delta_{j}|\mu_{scene},\sum_{scene}∼N(\mu_{scene},\sum_{scene})$$

Here, $\Delta_{j}$ denotes the offset for scenario j, $\mu_{scene}$ represents the mean offset across scenarios, and $\sum_{scene}$ denotes the covariance structure among scenarios. Parameters vary across scenarios according to a Gaussian distribution, allowing information sharing while retaining scenario-specific characteristics. Laboratory and field data exhibit consistent underlying trends but different fluctuation ranges; $\sum_{scene}$ captures this distinction.

### 3.3. Joint Modeling of Multiple Indicators and Uncertainty Quantification

#### 3.3.1. Joint Modeling of Multiple Indicators

Detection, recognition, tracking, and localization can exhibit correlated variation under shared imaging and environmental conditions. The joint model represents the four capability indicators through a multivariate distribution and a covariance structure:

(14) y|θ∼N(μ(θ),∑(θ))

The observation vector y comprises K = 4 capability indicators. Probability-type indicators are mapped to the real-valued domain by a logit transformation. The parameters describe the capability functions defined in Section 2, and the structured mean gives the expected value of each indicator under those parameters. The covariance matrix represents dependence among the indicators within the modeled groups.

The joint distribution compactly represents coupling and uncertainty covariance among the indicators and provides a statistical basis for joint prediction and confidence-interval estimation. When the number of indicators, K, is large, direct estimation of the full covariance matrix ∑ requires many degrees of freedom and can lead to overfitting. A shared latent-variable model reduces parameter dimensionality by representing the data through a few common driving factors:

(15) y=Wz+ε

Here, $z\in {\mathbb{R}}^{r}$ are the shared latent variables or common factors, where r≪K. These factors represent unobservable effects shared by all capability indicators, including those associated with scenario, platform, and imaging conditions. The latent variables are normally distributed z∼N(0,1). The matrix $W\in {\mathbb{R}}^{K\times r}$ is the factor loading matrix, whose K row describes the sensitivity of the K indicator to a common factor. Sparse or low-rank priors can be applied to suppress overfitting. The term ε∼N(0,Ψ) represents indicator-specific noise or unmodeled disturbances, where Ψ is usually a diagonal matrix representing the independent noise component of each indicator.

The mean function μ(θ) reflects the average behavior of each indicator. The covariance structure can be written as $\sum =WW^{T}+\Psi$, combining the contributions of common factors and indicator-specific noise. This factorization attributes inter-indicator correlations to a small set of common factors. Dimensionality reduction simplifies estimation by reducing the number of parameters. The resulting representation improves interpretability and predictive stability.

#### 3.3.2. Uncertainty Quantification and Calibration

Uncertainty quantification summarizes capability predictions through posterior distributions and uncertainty intervals. The model parameters are inferred from the observations, and posterior prediction describes the distribution of the capability indicators for a new input.

(1) Bayesian posterior sampling. The posterior distribution of a hierarchical Bayesian model generally has no closed-form analytical solution. Langevin dynamics sampling approximates this distribution through iterative updates:

(16) $$\theta^{\left(t+1\right)}=\theta^{\left(t\right)}+\varepsilon \nabla_{\theta }\log p(\theta |y)+\sqrt{2\varepsilon }\xi^{\left(t\right)}$$

Here, $\theta^{\left(t\right)}$ denotes the parameter value at iteration t, ε denotes the step size, or learning rate, and $\nabla_{\theta }\log p(\theta |y)$ denotes the gradient of the log-posterior density, which guides the parameters toward regions of higher posterior probability. The random noise term is denoted by $\xi^{\left(t\right)}∼N(0,1)$ and preserves stochasticity during sampling, enabling exploration of the parameter space. The method belongs to the stochastic gradient Langevin dynamics (SGLD) family and supports efficient inference on large-scale datasets.

The target log-posterior density is given by logp(θ|y)=logp(y|θ)+logp(θ)+C, which combines the data likelihood logp(y|θ) and the prior logp(θ). The prior term can encode the boundary, correlation, and task constraints specified in Equations (5)–(7). Probability-type indicators are sampled in logit space for numerical stability. Learning-rate annealing gradually reduces the step size to promote convergence.

(2) Posterior predictive distribution. After posterior samples ${\left\{\theta^{\left(m\right)}\right\}}_{m=1}^{M}$ are obtained, the capability indicators for a new scenario can be predicted. The posterior predictive distribution is computed using Monte Carlo approximation:

(17) $$p(y_{new}|y)=\int p(y_{new}|\theta )p(\theta |y)d\theta$$

A Monte Carlo approximation is applied to ${\left\{\theta^{\left(m\right)}\right\}}_{m=1}^{M}$, obtained from Equation (16).

(18) $$\hat{p}(y_{new}|y)\approx \frac{1}{M}\sum_{m}p(y_{new}|\theta^{\left(m\right)})$$

Equation (17) averages the conditional predictive probability of a new observation over all possible parameter values, weighted by the posterior distribution. Equation (18) approximates this quantity by Monte Carlo integration using posterior samples. The predictive probability is evaluated at each sampled parameter value, and the resulting values are averaged arithmetically.

(3) Calibration evaluation. Prediction interval coverage probability (PICP) is the proportion of observed values within a stated prediction interval. Prediction interval width (PIW) summarizes the average interval width. Coverage is assessed relative to the nominal level, with width interpreted alongside coverage. Expected calibration error (ECE) summarizes differences between predicted probabilities and observed frequencies. These quantities describe complementary aspects of calibration.

Predictive uncertainty can be expressed through the law of total variance as Var(*y*_new_|y) = E_θ_[Var(*y*_new_|*θ*, y)] + Var_θ_[E(*y*_new_|*θ*, y)]. This identity distinguishes conditional observation variability from variability in the conditional mean. The uncertainty summary uses four categories: observation noise, model parameters, scenario differences, and data-source differences. The source grouping refers to simulation, laboratory, field, and historical data; it does not identify individual equipment platforms.

## 4. Experimental Validation and Results Analysis

### 4.1. Experimental Setup and Dataset Construction

The scenario library contains marine, terrestrial, and aerial categories divided into twenty subcategories. Its intended coverage spans differences in background, viewing geometry, illumination, and environmental conditions. Table 4 lists the four data-source categories and their sample allocations.

**Figure 1:**
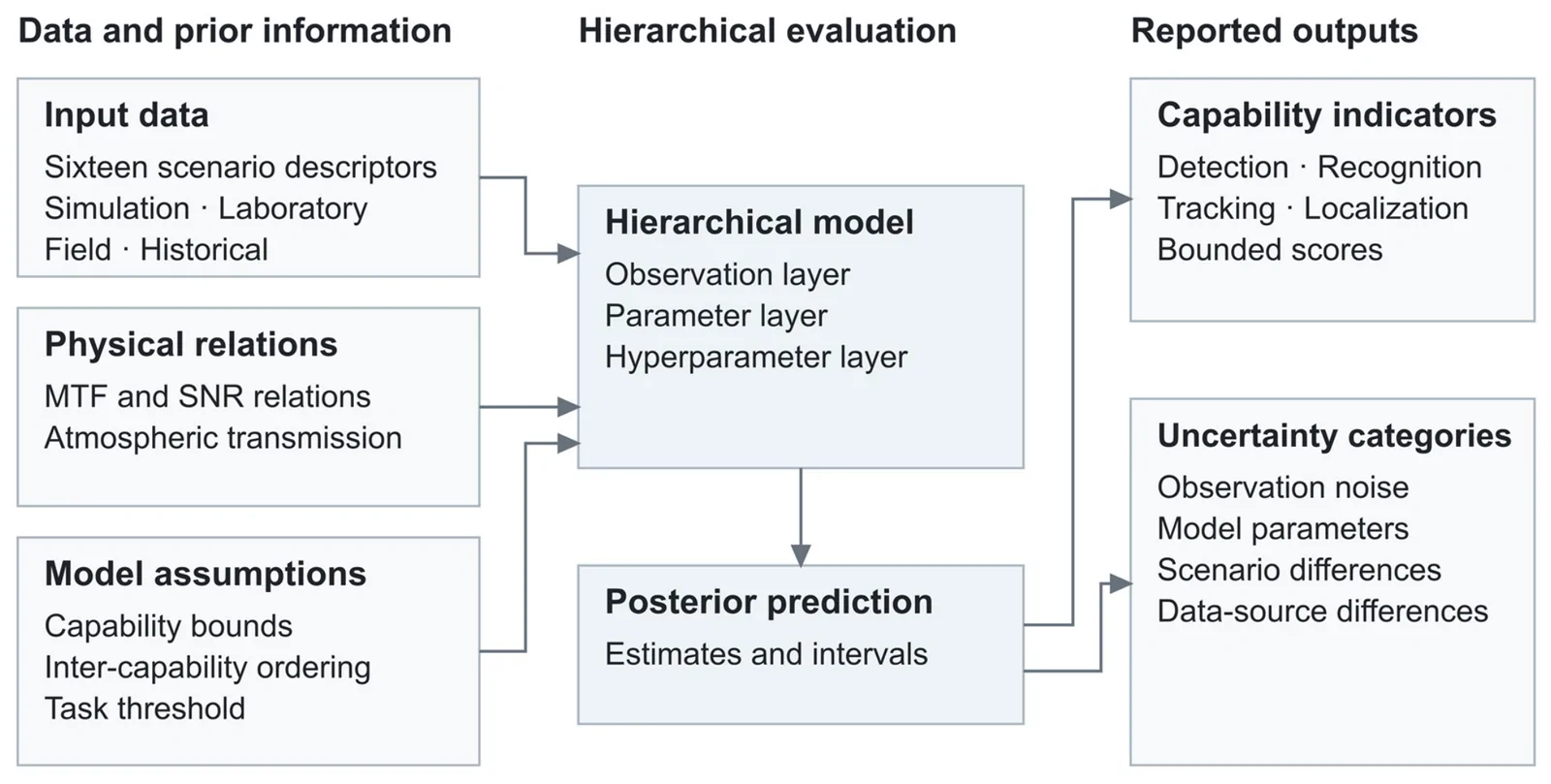
Organization of the evaluation framework. The left-hand blocks identify the input data, physical relations, and model assumptions; the central blocks show the hierarchical model and posterior prediction; the right-hand blocks identify the capability indicators and uncertainty categories. Arrows indicate the flow of information through the framework.

Figure 1 organizes the evaluation framework into input information, hierarchical evaluation, and reported outputs. The input-data block represents the sixteen scenario descriptors and the four data-source categories: simulation, laboratory, field, and historical observations. The physical-relations block summarizes the MTF, SNR, and atmospheric-transmission information described in Section 3.1.2. The model-assumptions block identifies the capability bounds, inter-capability ordering, and task threshold specified in Section 3.1.1. Arrows from these blocks indicate the information supplied to the hierarchical evaluation.

The central model block contains the three layers introduced in Section 3.2. The observation layer represents the four capability indicators and source-dependent noise; the parameter layer separates a shared baseline from scenario-specific offsets; and the hyperparameter layer describes variation among scenarios within the hierarchy. Posterior prediction yields estimates and intervals. The upper output branch reports the bounded detection, recognition, tracking, and localization scores. The lower branch lists the uncertainty categories used in the uncertainty summary: observation noise, model parameters, scenario differences, and data-source differences.

**Figure 2:**
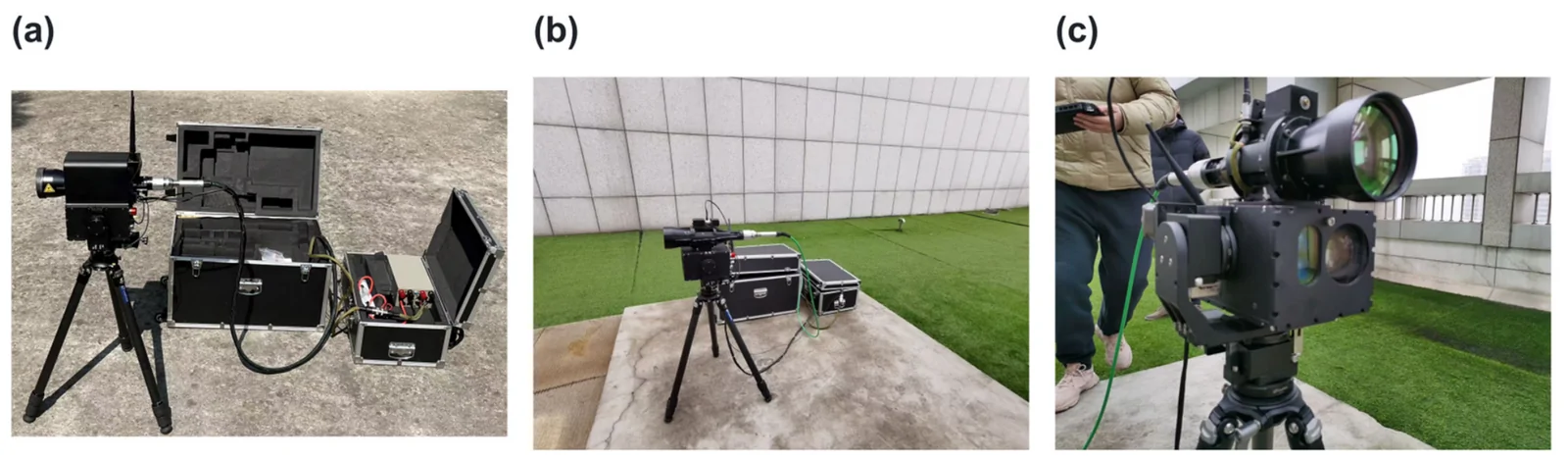
Photographs of the verification setup: (**a**) tripod-mounted payload, transport cases, external equipment, and connecting cables on pavement; (**b**) overall installation beside a tiled wall and artificial turf; (**c**) close view of the upper optical assembly and the front-panel optical windows.

Figure 2 documents the physical arrangement of the verification setup from three viewpoints. Panel (a) shows the tripod-mounted electro-optical payload, open transport cases, and cables connecting the payload to external equipment on pavement. Panel (b) provides an overall view of the installation beside a tiled wall and artificial turf. Panel (c) shows the upper cylindrical optical assembly and the two optical windows in the lower front panel. The tripod supports the mounted payload, and the connecting cables link it to the external equipment shown alongside the cases.

The imaging optics included a motorized zoom lens, model LD-LF12Z70A18M2 (Lingdong Optics, Shenzhen, China). The manufacturer specifies a focal-length range of 12–850 mm, a nominal zoom ratio of 70×, an F-number range of 2.8–10, a 1/1.8-inch image format, and motorized zoom and focus [25]. These catalog specifications identify the installed optics and are distinct from the scenario input ranges in Section 2.1.2.

The field test described here was conducted on 25 November 2021. Meteorological context for that date is provided by the NOAA Local Climatological Data, Version 2, records for XIANYANG station CHI0000ZLXY (34.447119° N, 108.751592° E; elevation 479.1 m) [26]. The 24-h records cover 00:00–23:00 local time (UTC+08:00). Across this calendar day, air temperature ranged from −3 to 15 °C (mean 4.42 °C), relative humidity from 27% to 93% (mean 65.5%), visibility from 5 to 8 km (mean 6.42 km), and wind speed from 0 to 2 m/s (mean 1.25 m/s). Mist was reported at 21:00–23:00. These station observations provide a regional reference for the test date.

The 4500-sample corpus is divided into training, validation, and test partitions of 3000, 800, and 700 samples. The training partition contains 2000 simulation, 400 laboratory, 120 field, and 480 historical samples. The validation partition contains 533 simulation, 67 laboratory, 55 field, and 145 historical samples. The test partition contains 467 simulation, 33 laboratory, 25 field, and 175 historical samples. These counts follow the source allocation in Table 4. Training is used for fitting, validation for model selection, and the test partition for the reported comparisons.

The comparison includes a mechanism-based baseline, data-driven methods, and hybrid combinations. Random forest [27], support vector regression [28], and a multilayer perceptron (MLP) are the data-driven comparators; weighted averaging and ensemble learning are the hybrid methods. The cross-scenario and sample-size comparisons use the MLP as the data-driven comparator.

All reported comparisons use the sixteen input descriptors and four capability outputs defined in Section 2. The mechanism-based baseline applies the capability relations, whereas the data-driven methods estimate the relationship between inputs and outputs. The ablation comparison evaluates the complete formulation with variants that omit the mechanism-informed priors, hierarchical structure, joint modeling, or uncertainty quantification, using the same prediction-error metrics.

### 4.2. Performance Evaluation and Comparative Analysis

Table 5 summarizes prediction error, explained variance, interference robustness, and prior contribution. In this comparison, the proposed method has an RMSE of 0.118, approximately 7.8% lower than the 0.128 of the best-performing alternative. Its MAE, R^2^, and robustness score are 0.089, 0.867, and 0.78, respectively. Prior contribution describes the influence of prior information; a larger value does not necessarily indicate a better model. The lower prediction errors do not imply superiority on every reported quantity.

**Figure 3:**
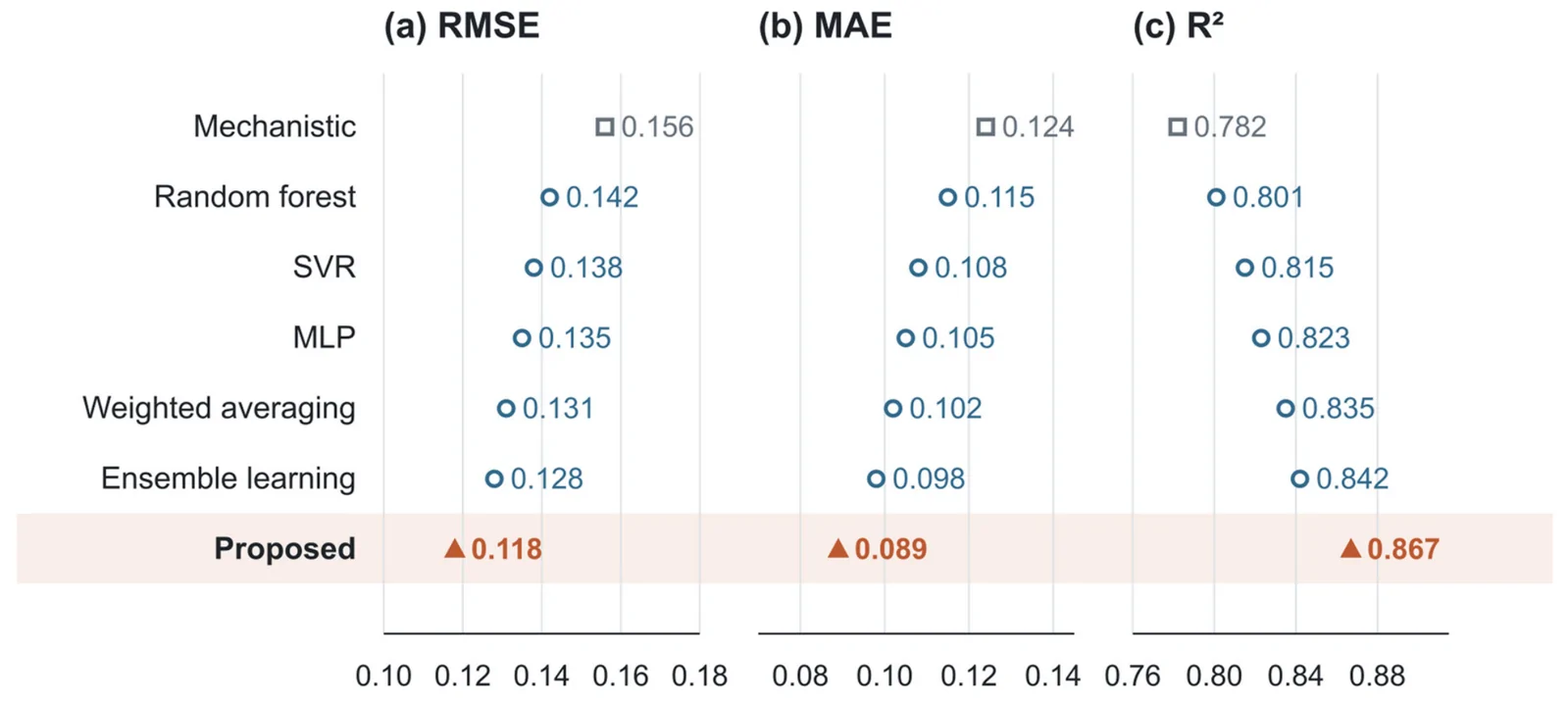
Prediction metrics for the seven methods in Table 5: (**a**) RMSE; (**b**) MAE; (**c**) R^2^. Each panel uses its own scale. SVR denotes support vector regression; MLP denotes multilayer perceptron. Open squares denote the traditional mechanism-based method, open circles denote the other comparator methods, and filled triangles denote the proposed method.

Figure 3 presents the RMSE, MAE, and R^2^ values from Table 5 in separate panels. Across the reported methods, the proposed method has the lowest RMSE and MAE and the highest R^2^.

Removing the mechanism-informed priors increases RMSE from 0.118 to 0.135, or approximately 14.4%, in the ablation comparison in Table 6. The variants without hierarchical structure and joint modeling have RMSE values of 0.128 and 0.125, respectively. These differences compare configurations within the same error metric; RMSE and MAE describe separate aspects of prediction error.

**Figure 4:**
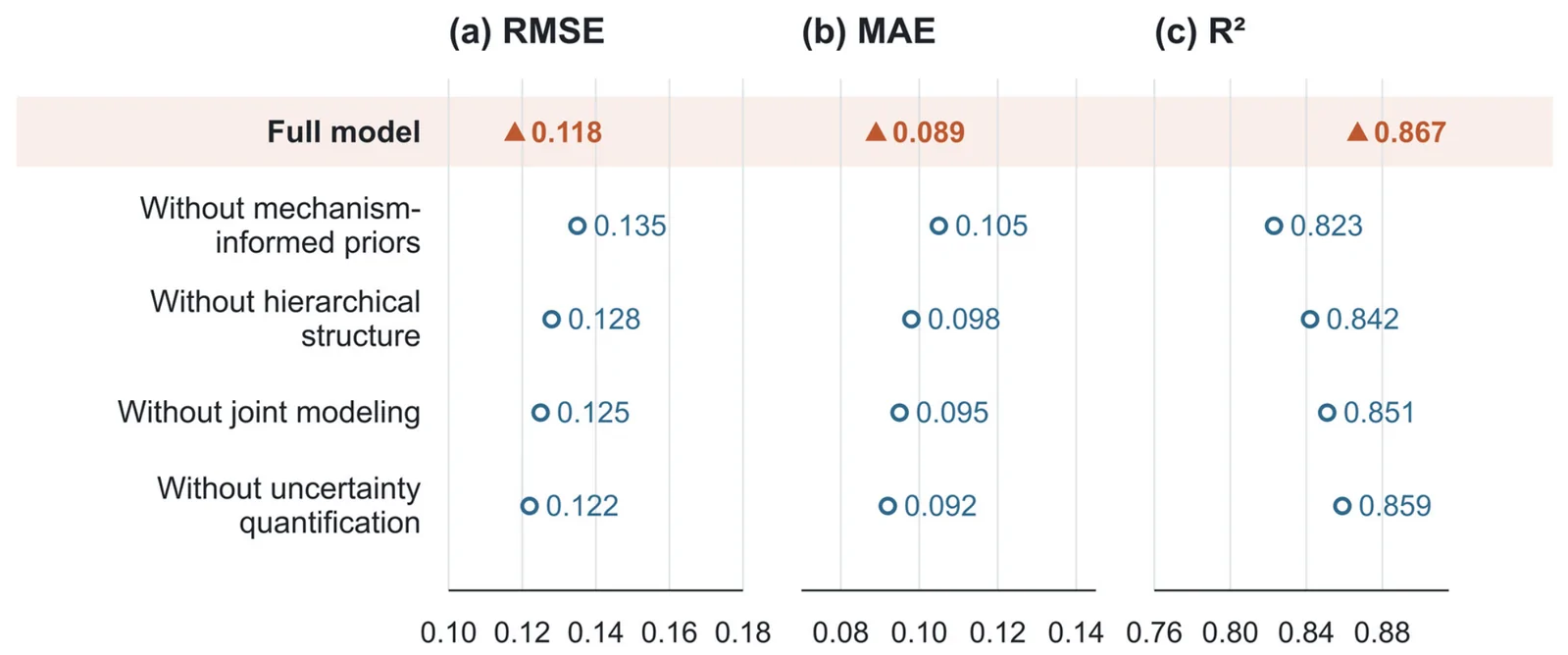
Ablation results from Table 6: (**a**) RMSE; (**b**) MAE; (**c**) R^2^. Each panel uses its own scale. Markers show the reported values for the full model and the four ablation configurations. Filled triangles denote the full model, and open circles denote the ablation configurations.

Across the ablation configurations in Figure 4, the complete model has the lowest reported RMSE and MAE and the highest R^2^. Omitting the mechanism-informed priors produces the largest RMSE increase, while the magnitude of the changes in the other variants depends on the component removed. These comparisons describe the individual ablations; they do not establish the effects of all possible interactions among components.

For the evaluation summarized in Table 7, observation noise contributes 35.2%, model parameters 28.7%, scenario differences 21.3%, and data-source differences 14.8%. These percentages describe the reported allocation across the four uncertainty categories.

**Figure 5:**
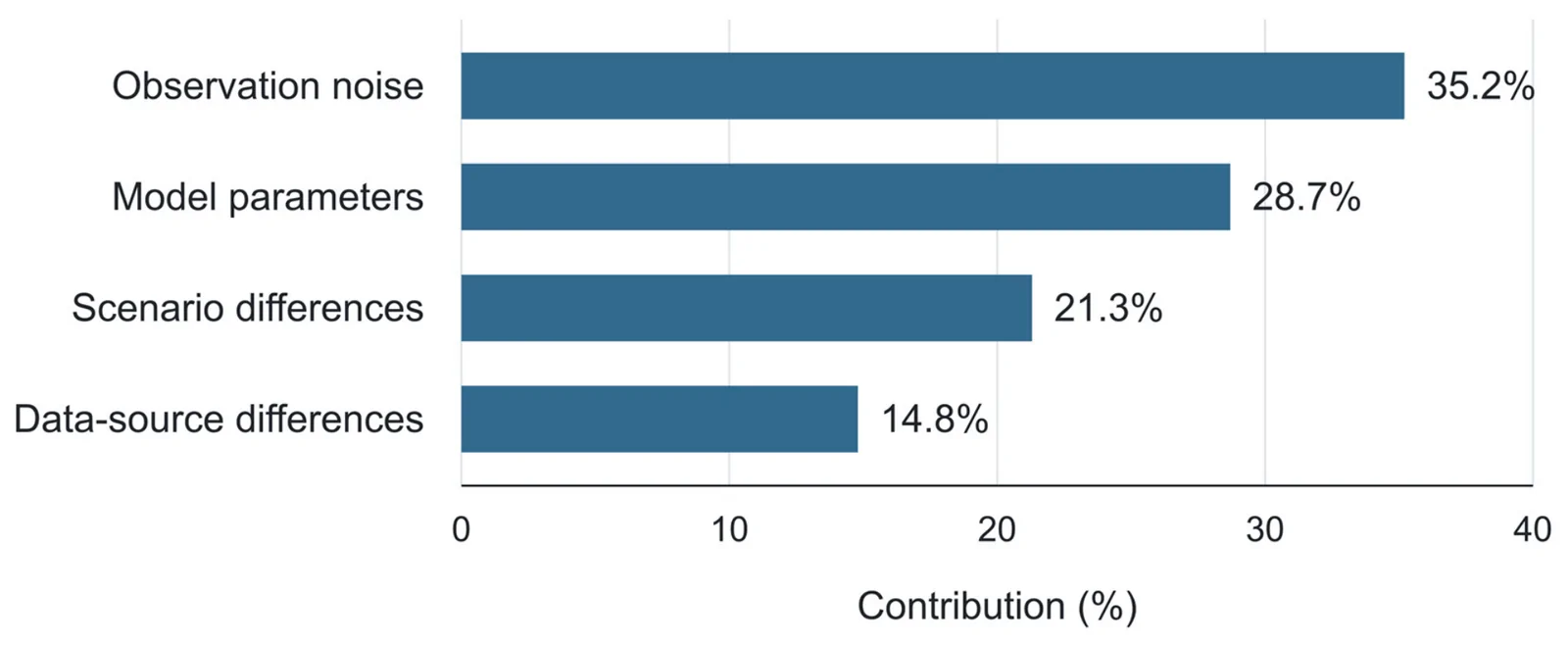
Contributions of the four uncertainty categories reported in Table 7. Percentages sum to 100%.

Observation noise and model-parameter uncertainty account for 63.9% of the contributions shown in Figure 5, with scenario and source differences accounting for the remaining 36.1%. The source category represents differences among simulation, laboratory, field, and historical observations, rather than independently identified effects of individual equipment platforms.

A reliability curve compares nominal and empirical coverage. It complements the prediction-error and interval summaries in Table 1 and Table 3.

The laboratory comparison gives a bias of −0.006, an RMSE of 0.129, and coverage of 0.943 for nominal 95% intervals. The sensitivity results in Table 2 rank SNR and atmospheric transmittance above the other listed factors. These aggregate summaries characterize deviations from ideal agreement and sensitivity to input conditions; they do not describe individual failed samples.

**Figure 6:**
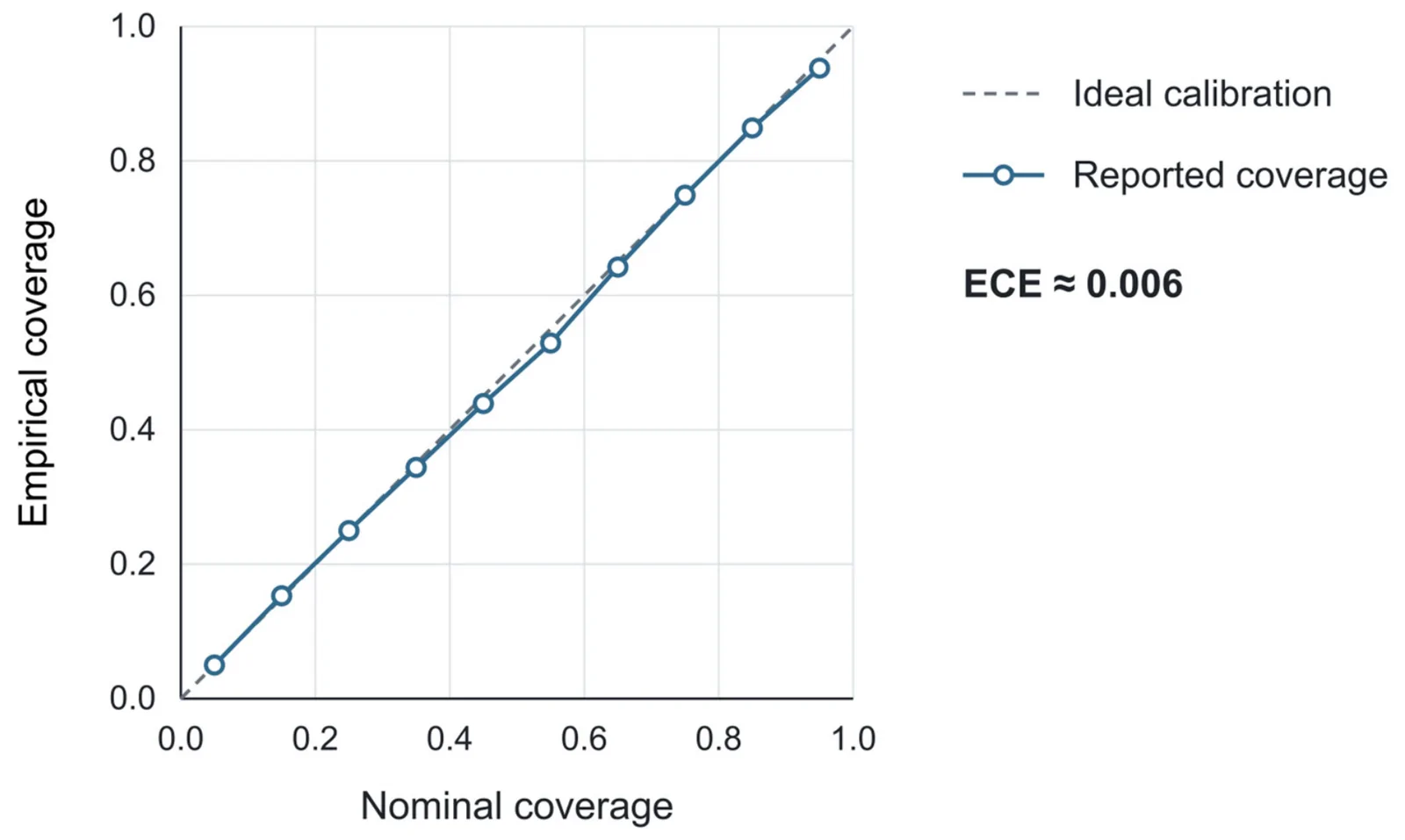
Reliability curve comparing nominal and empirical coverage at ten reported levels. The dashed line indicates ideal calibration; the reported ECE is approximately 0.006.

At the ten nominal coverage levels shown in Figure 6, the reported ECE is approximately 0.006. Table 1 contains the metrics for the separate simulation-validation evaluation.

### 4.3. Evaluation of Generalization Ability and Computational Complexity

The four cross-scenario tasks in Table 8 are marine to terrestrial, terrestrial to marine, daytime to nighttime, and nighttime to daytime. Lower values indicate smaller prediction errors within each transfer setting. The proposed method has the lowest reported error in all four tasks, with varying margins over the alternatives.

Performance under scenario changes is evaluated separately from performance in the pooled test partition. All three methods have higher reported errors for daytime-to-nighttime transfer than for the reverse direction. Generalization performance varies with the direction of transfer and cannot be represented by a single level across environments.

Across the four training set sizes in Table 9—100, 500, 1000, and 2000 samples—the proposed method has the lowest reported RMSE. At 100 samples, its RMSE is 0.165, compared with 0.198 for the MLP and 0.234 for the traditional method. The MLP-to-proposed RMSE ratio in the final column ranges from approximately 1.14 to 1.20. This ratio compares errors at a fixed sample size; it does not measure a reduction in the number of samples required.

**Figure 7:**
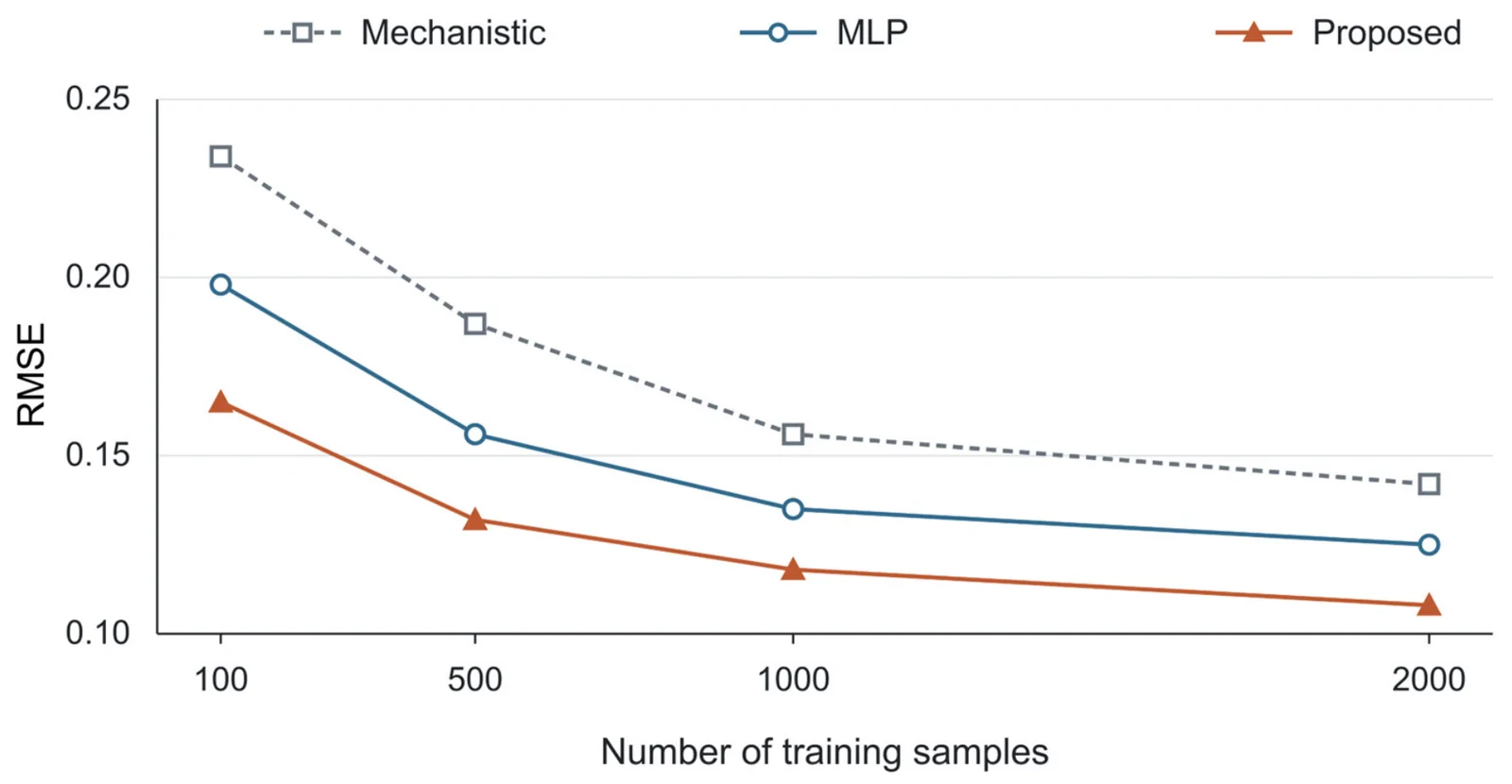
RMSE at the four training set sizes reported in Table 9. Markers indicate the reported values; straight lines connect adjacent values.

Errors decrease with increasing training set size for all three methods in Figure 7. Over the four reported sample sizes, the absolute RMSE gap between the proposed method and the MLP narrows from 0.033 at 100 samples to 0.017 at 2000 samples.

The reported computational comparison uses an Intel Core i9-13900K (Intel Corporation, Santa Clara, CA, USA) workstation with 64 GB of memory and an NVIDIA RTX 4090 GPU (NVIDIA Corporation, Santa Clara, CA, USA) with 24 GB of VRAM, running Ubuntu 22.04 (released on 21 April 2022), Python 3.10 (first released on 4 October 2021), and PyTorch 2.0 (released on 15 March 2023). Training time, inference time, and memory usage under this benchmark setting are listed in Table 10.

In Table 10, training takes 187.3 s for the proposed method, 156.7 s for SVR, and 234.5 s for the neural network. The proposed method’s inference time of 18.6 ms exceeds those of the other listed methods. It uses 134.7 MB of memory, compared with 156.8 MB for the neural network and 89.3 MB for SVR. These measurements describe the accuracy–cost trade-off within the reported benchmark and do not establish a universal latency requirement for deployment.

**Figure 8:**
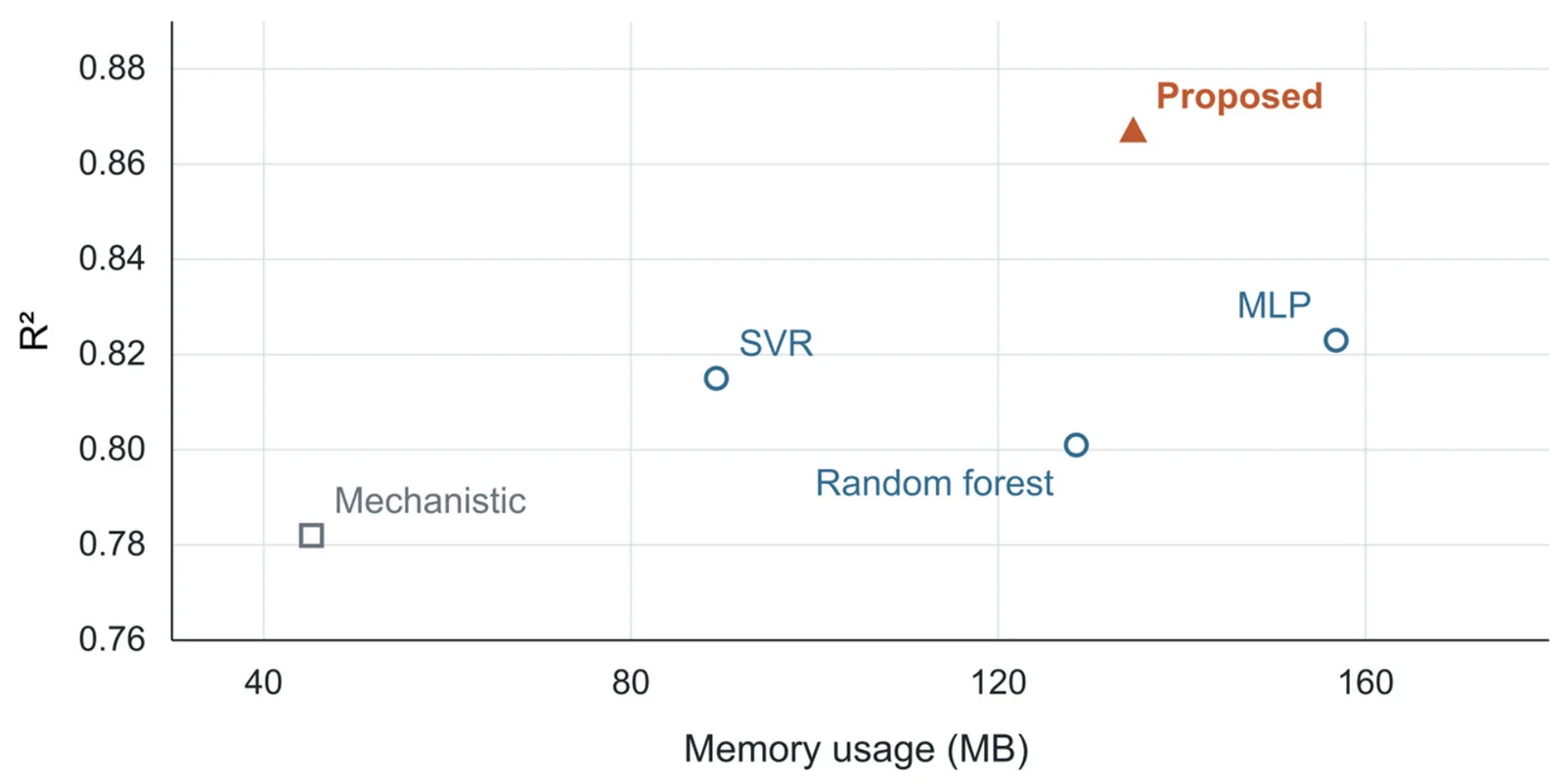
R^2^ versus memory usage for the five methods with values in both Table 5 and Table 10. SVR denotes support vector regression; MLP denotes multilayer perceptron. Open squares denote the traditional mechanism-based method, open circles denote random forest, SVR, and MLP, and filled triangles denote the proposed method.

The R^2^–memory comparison in Figure 8 uses the values in Table 5 and Table 10. The proposed method combines an R^2^ of 0.867 with memory usage of 134.7 MB. The traditional method requires less memory and has an R^2^ of 0.782, while the neural network uses 156.8 MB with an R^2^ of 0.823. These reported trade-offs do not imply a single preferred method under every resource constraint.

## 5. Conclusions

The hierarchical Bayesian framework evaluates detection, recognition, tracking, and localization capability by representing physical information and heterogeneous data within observation, parameter, and hyperparameter layers. The reported evaluation covers prediction error, calibration, ablation settings, cross-scenario performance, training set size, and computational cost. In the overall comparison, the proposed method has an RMSE of 0.118, an MAE of 0.089, and an R^2^ of 0.867. These findings apply under the stated data and model assumptions.

Interpretation depends on the simplified capability functions, the composition of the data sources, and the computational setting. With only 25 field samples in the 700-sample test partition, pooled performance does not constitute comprehensive field validation. Evaluation on independently collected datasets would help define the range over which the reported relationships remain applicable.

## Figures and Tables

**Table 1:** Overall results of simulation-based validation (*n* = 1000 scenarios).

Metric	Value
Mean absolute percentage error (fraction)	0.068
RMSE	0.121
MAE	0.095
R^2^	0.873
PICP (95%)	0.948
PIW (95%)	0.186
ECE	0.021

**Table 2:** Sensitivity analysis of simulation parameters.

Rank	Factor	Symbol	First-Order Sensitivity ($S_{i}$)	Total Sensitivity- ($S_{T_{i}}$)
1	Signal-to-noise ratio	SNR	0.36	0.48
2	Atmospheric transmittance	$\tau_{atm}$	0.22	0.31
3	Target feature/contrast	$\theta_{t\arg et}$	0.18	0.27
4	Background clutter intensity	$\sigma_{clutter}$	0.12	0.19
5	Platform pointing stability	$\sigma_{platform}$	0.07	0.13

**Table 3:** Semi-physical validation results.

Metrics	Value
Correlation coefficient	0.92
RMSE	0.129
MAE	0.101
Bias	−0.006
PICP (95%)	0.943
PIW (95%)	0.194

**Table 4:** Sample allocation by source and partition.

Data Source	Total	Train/Val/Test	Obs. Noise Sigma_s
Physics simulation (LHS)	3000	2000/533/467	0.020
Semi-physical lab experiments	500	400/67/33	0.035
Field campaigns (real payload)	200	120/55/25	0.070
Historical mission records	800	480/145/175	0.055
Total	4500	3000/800/700	--

**Table 5:** Overall performance comparison results.

Method	RMSE	MAE	R^2^	Robustness to Interference	Prior Contribution
Traditional Mechanism-Based Method	0.156	0.124	0.782	0.65	0.95
Random Forest	0.142	0.115	0.801	0.58	0.12
Support Vector Machine	0.138	0.108	0.815	0.62	0.08
Neural Network	0.135	0.105	0.823	0.55	0.05
Weighted averaging	0.131	0.102	0.835	0.68	0.25
Ensemble learning	0.128	0.098	0.842	0.71	0.18
Proposed method	0.118	0.089	0.867	0.78	0.42

**Table 6:** Ablation study results.

Configuration	RMSE	MAE	R^2^
Full Model	0.118	0.089	0.867
Without Mechanism-Informed Priors	0.135	0.105	0.823
Without Hierarchical Structure	0.128	0.098	0.842
Without Joint Modeling	0.125	0.095	0.851
Without Uncertainty Quantification	0.122	0.092	0.859

**Table 7:** Uncertainty decomposition results.

Sources of Uncertainty	Contribution (%)
Observation Noise	35.2
Model Parameters	28.7
Scenario Differences	21.3
Data-source differences	14.8

**Table 8:** Cross-scenario transfer performance.

Source Scenario → Target Scenario	Traditional Method	Data-Driven (MLP)	Proposed Method
Marine → Terrestrial	0.156	0.142	0.125
Terrestrial → Marine	0.162	0.148	0.128
Daytime → Nighttime	0.178	0.165	0.138
Nighttime → Daytime	0.145	0.132	0.115

**Table 9:** Small-sample learning performance.

Number of Training Samples	Traditional Method	Data-Driven (MLP)	Proposed Method	MLP/Proposed RMSE
100	0.234	0.198	0.165	1.20
500	0.187	0.156	0.132	1.18
1000	0.156	0.135	0.118	1.14
2000	0.142	0.125	0.108	1.16

**Table 10:** Comparison of computational complexity.

Method	Training Time (s)	Inference Time (ms)	Memory Usage (MB)
Traditional mechanistic methods	12.5	2.3	45.2
Random Forest	89.3	15.7	128.5
Support Vector Machine	156.7	8.9	89.3
Neural Network	234.5	12.4	156.8
Proposed method	187.3	18.6	134.7

## Data Availability

The data presented in this study are available from the corresponding author upon reasonable request.
